# Decoding epitranscriptomic regulation of viral infection: mapping of RNA *N*^6^-methyladenosine by advanced sequencing technologies

**DOI:** 10.1186/s11658-024-00564-y

**Published:** 2024-03-27

**Authors:** Xiangdong Fan, Yitong Zhang, Ruiying Guo, Kuo Yue, Guy Smagghe, Yongyue Lu, Luoluo Wang

**Affiliations:** 1 National Key Laboratory of Green Pesticide, College of Plant Protection, South China Agricultural University, Guangzhou, 510642 China; 2 Molecular and Cellular Life Sciences, Department of Biology, Vrije Universiteit Brussel (VUB), 1050 Brussels, Belgium; 3 Institute of Entomology, Guizhou University, Guiyang, 550025 China; 4 Department of Plants and Crops, Faculty of Bioscience Engineering, Ghent University, 9000 Ghent, Belgium

**Keywords:** m^6^A modification, Epitranscriptome sequencing technologies, Viral infection

## Abstract

Elucidating the intricate interactions between viral pathogens and host cellular machinery during infection is paramount for understanding pathogenic mechanisms and identifying potential therapeutic targets. The RNA modification *N*^6^-methyladenosine (m^6^A) has emerged as a significant factor influencing the trajectory of viral infections. Hence, the precise and quantitative mapping of m^6^A modifications in both host and viral RNA is pivotal to understanding its role during viral infection. With the rapid advancement of sequencing technologies, scientists are able to detect m^6^A modifications with various quantitative, high-resolution, transcriptome approaches. These technological strides have reignited research interest in m^6^A, underscoring its significance and prompting a deeper investigation into its dynamics during viral infections. This review provides a comprehensive overview of the historical evolution of m^6^A epitranscriptome sequencing technologies, highlights the latest developments in transcriptome-wide m^6^A mapping, and emphasizes the innovative technologies for detecting m^6^A modification. We further discuss the implications of these technologies for future research into the role of m^6^A in viral infections.

**Graphical Abstract:**

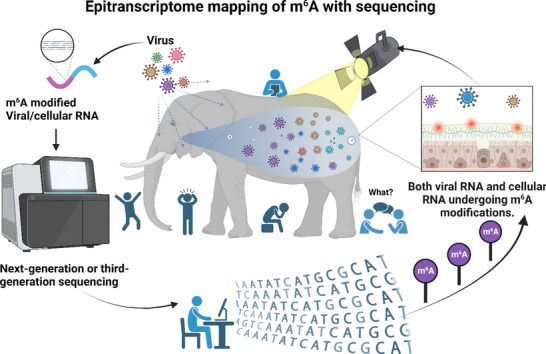

## Introduction

Throughout history, viruses, ranging from the common flu to emerging novel viruses, have imposed a substantial burden on public healthcare and resulted in significant economic and humanitarian losses [1, 2]. The recent coronavirus disease 2019 (COVID-19) pandemic, caused by the novel severe acute respiratory syndrome coronavirus 2 (SARS-CoV-2), serves as a stark reminder of the pressing need to unravel the intricate interactions between viruses and host cellular processes [2]. A spotlight was cast on RNA, particularly messenger RNA (mRMA), during this pandemic. As the primary component of the COVID-19 vaccine, it has been instrumental in saving millions of lives [3, 4]. In recognition of this monumental achievement, the 2023 Nobel Prize in Physiology or Medicine was awarded to Katalin Karikó and Drew Weissman for their pioneering work on mRNA vaccine technology. Their discoveries concerning nucleoside base modifications were crucial in developing effective mRNA vaccines against COVID-19. This significant medical advancement has been recognized as transforming the path of the pandemic and saving millions of lives. Moreover, the significance of RNA extends far beyond its application in vaccine technology. With its structure composed of the four canonical ribonucleotides—adenine (A), cytosine (C), guanine (G), and uracil (U)—and further complexity enriched with over 170 posttranscriptional chemical modifications observed across all life forms [5], RNA plays a crucial role in shaping cellular identity and directing a vast array of biological processes. Consequently, this brings to the fore the importance of comprehending RNA modifications, as they have far-reaching implications in both health and disease [6–9]. For instance, complementing our understanding of RNA modifications in host–pathogen interactions, we previously presented a pioneering study on spleen mRNA m^6^A methylation in response to malaria parasite infection [10]. Our findings demonstrated that m^6^A modifications serve as transcriptome-wide markers significantly influenced by the presence of *Plasmodium yoelii*, thereby reprogramming host immune responses and altering gene expression [10]. This work provides invaluable insights into the epitranscriptomic mechanisms that underlie pathogen-induced alterations in host biology.

**Fig. 1:**
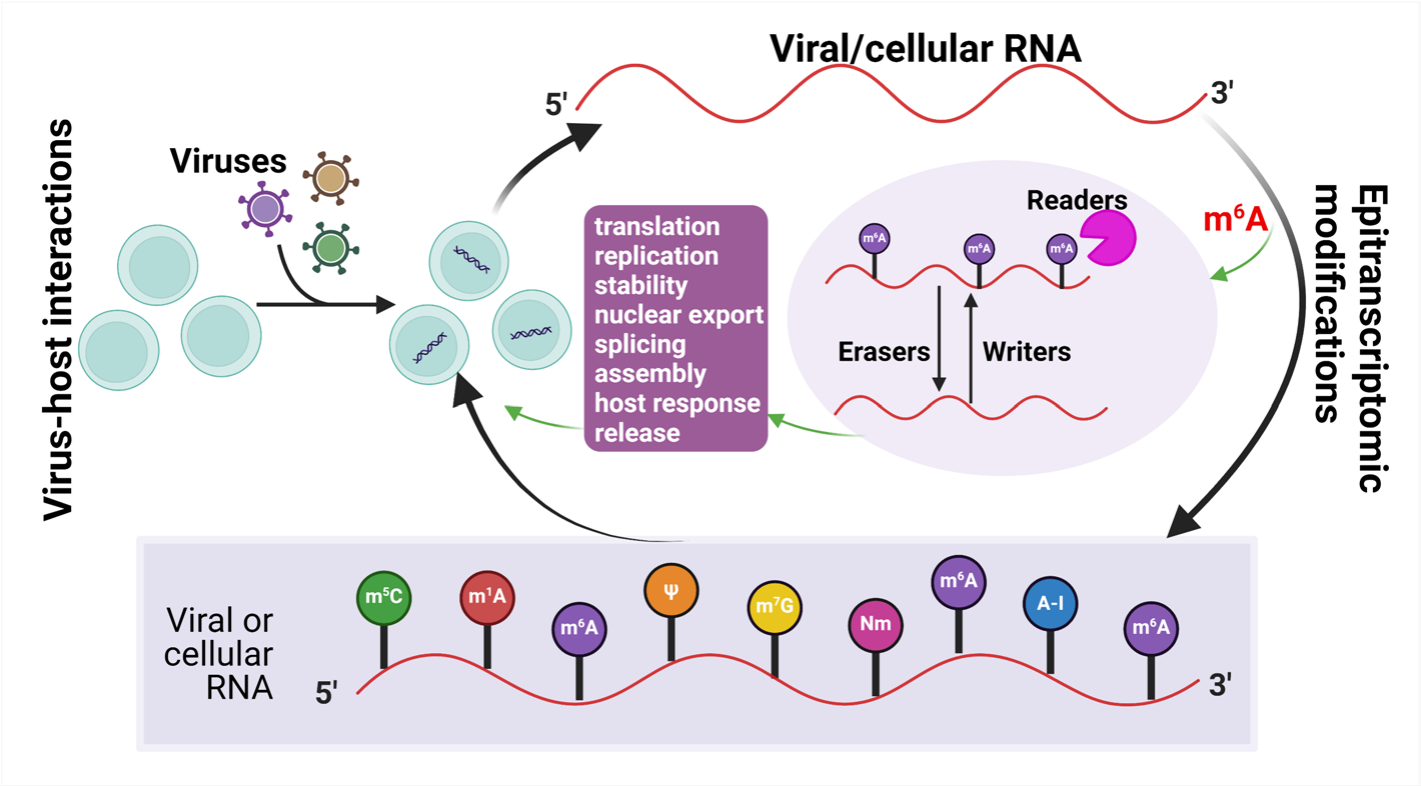
Overview of epitranscriptomic modifications in viral infections. This schematic represents the interplay between viral infection and RNA modifications. The top half of the figure illustrates the process of viral infection leading to the modification of both viral and cellular RNA, encompassing key RNA processes such as translation, replication, stability, nuclear export, splicing, viral assembly, host response, and viral release. The role of m^6^A modification is highlighted, showing its addition (writers), recognition (readers), and removal (erasers). The bottom half details various modifications found on viral and cellular RNA, including m^6^A, A-I (adenosine-to-inosine editing), m^1^A, ψ (pseudouridine), m^7^G, Nm (2′-o-methylation), and m^5^C, indicating the diverse epitranscriptomic landscape that viruses and cells navigate during infection. The figure was created using BioRender (BioRender.com)

RNA modifications, termed RNA epigenetics or epitranscriptomics, are critical regulatory elements that affect both cellular and viral RNA [11]. These chemical makers, as shown in Fig. 1, by influencing the RNA’s density, play a crucial role during viral infections [12]. In recent years, numerous RNA modifications have been reported. Among these most common dynamic modifications such as pseudouridine (Ψ), *N*^1^-methyladenosine (m^1^A), *N*^7^-methylguanosine (m^7^G), 5-methylcytidine (m^5^C), adenosine-to-inosine editing (A-to-I editing), and notably *N*^6^-methyladenosine (m^6^A)—the primary focus of this review [13]. For instance, research by Li et al. [14] has demonstrated that METTL3, an RNA methyltransferase, regulates viral m^6^A RNA modification and the host cell’s innate immune responses during SARS-CoV-2 infection. Complementary studies by Liu et al. [15] have further substantiated that both the genomic RNA and the negative-sense RNA of SARS-CoV-2 undergo dynamic m^6^A modification in human and monkey cells, indicating a significant role for m^6^A in the viral replication cycle [16]. Our depth of knowledge in epitranscriptomics is largely attributed to studies on m^6^A, which was first identified in cellular RNAs in the 1970s [17, 18]. This seminal work paved the way for the discovery of m^6^A in viral RNAs [19, 20], including the influenza A virus (IAV) exhibiting similar m^6^A levels to cellular RNA. Over the decades, the multifaceted role of m^6^A in regulating gene expression, especially its influence on various stages of viral lifecycle and host–virus interactions, has been gradually elucidated [21]. A detailed description of this topic is beyond the scope of this review, and we refer interested readers to some recent comprehensive reviews [11, 12, 16, 22].

**Fig. 2:**
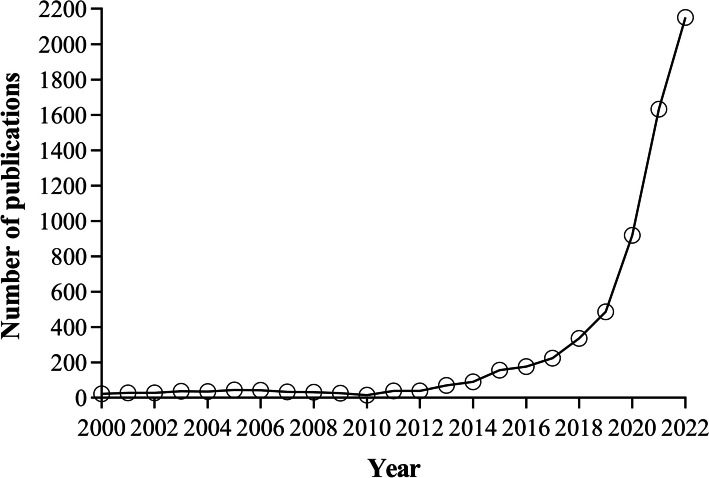
Number of publications containing the keywords “*N*^6^-methyladenosine” or “m^6^A” in the title or abstract indexed in the PubMed database from 2000 to 2022. The marked upsurge in publication volume, particularly noticeable from 2012 onward, correlates with the widespread adoption of advanced sequencing technologies, which have significantly enhanced the detection and study of m^6^A modifications

Mapping m^6^A distribution is essential for understanding this important marker in pathogenesis and many other essential biological processes. Yet for decades, the true regulatory potential and distribution of m^6^A remained poorly understood [23–25]. A breakthrough occurred in 2012 with the development of the methylated RNA immunoprecipitation sequencing (MeRIP-seq or m^6^A-seq) method for mapping the m^6^A methylome [23, 24], which rejuvenated the field of RNA modifications (Fig. 2). In today’s era of rapid development of next-generation sequencing (NGS) and third-generation sequencing (TGS) technologies, sequencing-based methods for mapping m^6^A modifications have progressed considerably [26, 27]. MeRIP-seq/m^6^A-seq and enhanced iterations of this technique have been pivotal in identifying cellular and viral RNAs containing m^6^A. Recent techniques, emphasizing high-resolution (e.g., base-level resolution) and quantitative sequencing techniques [28–30], offering a more comprehensive understanding of m^6^A modifications. This review, therefore, concentrates on recent advances in sequencing-based m^6^A mapping, addressing both the potential and challenges of current tools. We also highlight the future applications of m^6^A transcriptomic mapping during viral infections and explore future research directions regarding the involvement of m^6^A in viral infections. While m^6^A is our primary focus, it is noteworthy that other RNA modifications, such as 2′*O* methylation (Nm) [31] and terminal uridylation [32], have also been identified in viral RNAs, suggesting their potential roles in viral infections. For a more in-depth exploration of these modifications, readers are directed to recent reviews [11, 22, 33].

## Tracing the advancements: the evolutionary journey of m^6^A mapping technologies

**Fig. 3:**
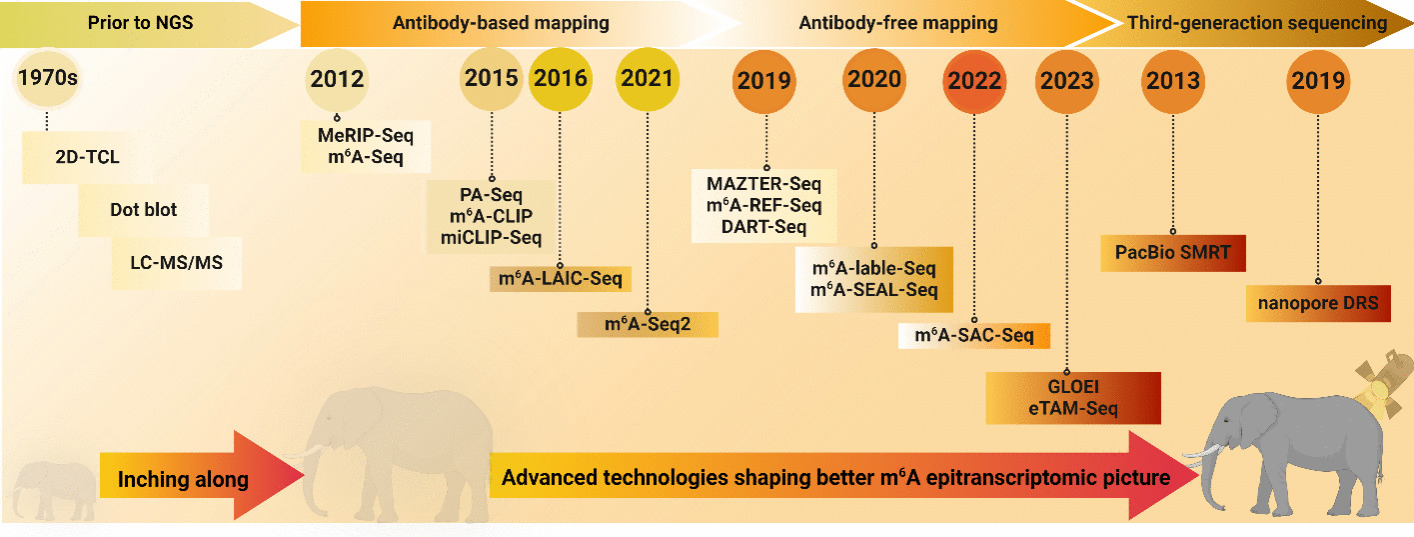
Chronological advancements in m^6^A epitranscriptomic sequencing technologies and an art metaphor (elephant and spotlight). Overview of the progressions of next-generation sequencing (antibody based and antibody free) and third-generation sequencing techniques developed over time for detecting m^6^A modifications. This figure employs visual metaphors, such as elephants and a spotlight, to suggest the significant impact and advanced nature of these technologies. The figure was created using BioRender (BioRender.com)

As the saying goes, “Sharpen the knife before cutting the wood,” the advancement of m^6^A detection techniques is fundamental for studying m^6^A functions. As depicted in Fig. 3, the journey of m^6^A mapping technologies is a testament to the dedication of the scientific community to understanding this crucial RNA modification.

### Early endeavors and limitations: prior to NGS

In the nascent stages of m^6^A research (i.e., before the advent of advanced sequencing technologies), m^6^A was primarily detected through bulk measurements for the global changes in RNA m^6^A levels, such as two-dimensional thin-layer chromatography (2D-TLC) [34], dot blot assays [10], and liquid chromatography–tandem mass spectrometry (LC–MS/MS) [35]. These techniques, while effective in detecting the presence and quantification of m^6^A modifications, offered little in terms of sequence-specific localization [36]. Additionally, techniques such as immunofluorescence and immunoprecipitation, though providing a more targeted approach, still lacked the resolution required for precise mapping. Hence, these early techniques, while laying the groundwork, highlighted the critical need for more sophisticated technologies. They brought into sharp focus the significance of m^6^A modifications, catalyzing a demand for innovation that could provide precise and comprehensive localization within the RNA sequence [23–25].

### Breakthrough in m^6^A mapping: advent of NGS-based approaches

The integration of NSG with m^6^A-specific immunoprecipitation marked a major leap forward in RNA biology. This combination, which led to the development of methodologies such as m^6^A-seq or MeRIP-seq [23, 24], allows for high-throughput detection of m^6^A modifications across the transcriptome. Since the development of these groundbreaking methodologies, the scope of research in RNA biology has broadened significantly (Figs. 2 and 3). This expansion has given rise to three principal strategies for comprehensive detection of RNA modifications, each offering unique insights and capabilities: (i) immunoprecipitation-based methods, such as MeRIP-seq and m^6^A-seq, utilize antibodies that specifically bind to the modified ribonucleotide. Subsequent developments, including photo-crosslinking-assisted m^6^A-seq (PA-m^6^A-seq)[37], m^6^A crosslinking immunoprecipitation sequencing (m^6^A-CLIP)/m^6^A individual-nucleotide resolution UV crosslinking and immunoprecipitation (miCLIP) [28, 38], m^6^A-level and isoform-characterization sequencing (m^6^A-LAIC-seq) [39], and m^6^A-seq2 [40], have further refined the process, aiming to enhance the specificity and resolution of m^6^A mapping. (ii) Approaches such as deamination adjacent to RNA modification targets sequencing (DART-seq) [41], MAZTER-seq [42], m^6^A-sensitive RNA-endoribonuclease-facilitated sequencing (m^6^A-REF-seq) [43], and evolved TadA-assisted *N*^6^-methyladnosine sequencing (eTAM-seq) [44] use specific enzymes to selectively distinguish modified and unmodified bases. Additionally, (iii) chemical-assisted methods, such as FTO-assisted m^6^A selective chemical labeling method (m^6^A-SEAL) [32], m^6^A-label-seq [45], m^6^A selective allyl chemical labeling and sequencing (m^6^A-SAC-seq) [30], and glyoxal and nitrite-mediated deamination of unmethylated adenosines (GLORI) [46], have also emerged and offer new avenues for m^6^A research (Table 1). These approaches are similar in that they isolate the RNA after inducing changes to the surrounding nucleotides, followed by reverse transcription and short-read cDNA sequencing to detect these changes.

**Table 1:** Comparison of current m^6^A transcriptome-wide profiling techniques

Strategy	Technique	Principle	Resolution	Starting materials	Sensitivity	Specificity	Stoichiometry	Advantages	Limitations	References
Antibody based	MeRIP-seq/m^6^A-seq	IP by m^6^A antibody; NGS	100–200 nt	2–400 μg mRNA	Medium	Medium	No	First method for global mapping of m^6^A, applicable, widely adopted;	Nonspecific antibody interactions; low resolution; without stoichiometry	[23, 24]
	PA-m^6^A-seq	IP by m^6^A antibody; photo-crosslinking; NGS	~ 23 nt	10 μg mRNA	Medium	Medium	No	Higher resolution	Live cells required, does not discriminate m^6^A from m^6^Am, nonspecific antibody interactions, without stoichiometry	[37]
	m^6^A-CLIP/miCLIP	IP by m^6^A antibody; photo-crosslinking; NGS	1 nt	20 μg mRNA	High	High	No	Single-nucleotide resolution	Nonspecific antibody interactions, without stoichiometry	[28, 38]
	m^6^A-LAIC-seq	Full-length RNA IP by m^6^A antibody; NGS	1500 nt	150 μg total RNA	Medium	Medium	Yes	Quantifying m^6^A stoichiometry	Does not differentiate adjacent m^6^A sites, low resolution	[39]
	m^6^A-seq2	m^6^A-IP on the pooled samples; NGS	100–200 nt	1.2/*n* μg mRNA per sample (*n* = total sample number)	Medium	Medium	No	Allows quantification across genes and samples; lower technical/batch variability, starting material, and costs	Nonspecific antibody interactions, low resolution, without stoichiometry	[40]
Chemical assisted	m^6^A-SEAL-seq	Methyl oxidation by FTO; NGS	100–200 nt	5 μg mRNA	High	High	No	Low input material, higher sensitivity	Low resolution, complicate procedures, without stoichiometry	[52]
	m^6^A-label-seq	Metabolic labeling of m^6^A site; NGS	1 nt	5 μg total RNA	Medium	Medium	No	Single-nucleotide resolution	Live cells required, does not discriminate m^6^A from m^6^Am, without stoichiometry	[29]
	m^6^A-SAC-seq	MjDim1 treatment; NGS	1 nt	2–50 ng mRNA	High	low	N/A	Single-nucleotide resolution, quantitative tracking of m^6^A	Sequence preference, low specificity and efficiency	[53]
	GLORI	Glyoxal and nitrite to deaminate unmethylated A to I; NGS	1 nt	100 ng mRNA	High	High	Yes	Single-nucleotide resolution, able to define m^6^A clusters, stoichiometric	High sequencing cost, extra procedures needed to discriminate m^6^A from some other A modifications	[46]
Enzyme assisted	DART-seq	Deamination adjacent to m^6^A modification targets; NGS	1 nt	10 ng to 1 μg total RNA	Low	Low	No	Single-nucleotide resolution, stoichiometric	Live cells required	[41]
	m^6^A-REF-seq	MazF RNase digestion; NGS	1 nt	100 ng mRNA	High	High	Yes (Only ACA)	High resolution, antibody free, stoichiometric	Only cover ACA motif	[43]
	MAZTER-seq	MazF RNase digestion; NGS	1 nt	100 ng mRNA	High	High	Yes (Only ACA)	High resolution, antibody free, stoichiometric	Only cover ACA motif	[42]
	eTAM-seq	Global A deamination by TadA, RT–PCR, and Sanger sequencing	1 nt	259 pg mRNA (10 cells)	High	High	Yes	Stoichiometric, much lower input requirement, deep-sequencing free	Require control transcriptome, less sensitive to sites of low methylation levels	[44]
TGS	PacBio SMRT	SMRT	1 nt	N/A	Low	Low	Yes	Long-read sequencing on native RNA	Not been further developed or commercialized	[55]
	Nanopore DRS	Nanopore DRS	1 nt	500 ng to 1 μg mRNA	Low	Low	Yes	Long-read sequencing on native RNA, able to detect other modifications as well as m^6^A	High cost, low accuracy, high requirements of the quantity and quality of input RNA, require a low or no methylation control	[40, 57]

Despite the transformative impact of NGS-based methodologies in charting the landscape of RNA modifications transcriptome wide, they come with inherent limitations. One significant challenge is their reliance on the availability of modification-specific antibodies, chemical compounds, or specific signatures resulting from reverse transcription (RT), which have certain limitations, such as (i) the cross reactivity or low sensitivity of antibodies or chemical reactions, (ii) biases induced by the complicated multistep experimental protocols (e.g., m^6^A-SEAL-seq), and (iii) often do not provide single-nucleotide resolution or the ability to identify modifications on individual RNA molecules (such as the most widely used MeRIP-seq).

### Enter the third generation: the next frontier

Before discussing the integration of TGS in the study of m^6^A RNA modifications, it is also important to acknowledge the increasing attention on RNA viruses, highlighted by these newly emerging viruses of the twenty-first century (COVID-19, Zika, Ebola, acquired immunodeficiency syndrome (AIDS), Middle East respiratory syndrome (MERS), SARS, Avian flu, etc.) [47]. As taught by SARS-Cov-2, which has infected more than half of the world’s population, RNA viruses are known for their rapid replication and mutation rates, which presents significant challenges in early detection and effective management of outbreaks. The current detection methods, such as NGS-based cDNA sequencing, often fall short of providing complete information on the viral genome [26]. From this perspective, the TGS, on the other hand, offers a more accurate approach and is more in line with the context of the era.

In the early 2010s (shortly after the appearance of NGS), TGS technologies emerged. The initial authentic TGS technology, known as “single-molecule real-time” (SMRT) sequencing, was launched by Pacific Biosciences (PacBio) in 2011 [48]. Subsequently, in 2014, Oxford Nanopore Technologies (ONT) introduced the nanopore sequencing technology [49]. The most distinguishing features of TGS are single-molecule sequencing and sequencing in real time (as opposed to NGS, where sequencing is paused after each base incorporation) [50]. The capacity to generate long reads is the distinctive advantage of both technologies, overcoming all known difficulties of conventional short-read sequencing and allowing to characterize complex genomic regions [51]. This advancement also significantly facilitated the identification of full-length transcripts and the direct detection of RNA modifications (without the need for amplification or fragmentation) on native RNA molecules, marking a transformative step forward in epitranscriptomics [26]. However, this approach still faces challenges in terms of precision, accuracy, and the need for improved computational tools. As the field continues to evolve, further advancements in TGS are expected to enhance our understanding of m^6^A modifications and their roles in various biological processes.

To conclude, just as we aim to simulate this historical process in Fig. 3, the evolution of m^6^A mapping technologies can be likened to the story of the blind men and the elephant. Just as each blind man had a limited perception based on the part of the elephant they touched, early techniques in RNA modification research provided only partial insights into the complex landscape of m^6^A modifications. However, as technology advanced, our understanding became increasingly comprehensive, akin to gradually gaining a clearer and more holistic view of the elephant. While we currently stand at a particular stage in this historical progression, the future holds the promise of continued advancements. We believe that forthcoming technological innovations will act like a spotlight, illuminating the critical functions and mechanisms of m^6^A modifications, as well as their roles in the process of viral infections. This ongoing journey of discovery and understanding in the field of RNA biology is an exciting and evolving narrative, where each new technology brings us closer to a complete understanding of the intricate world of m^6^A modifications. Hence, in the forthcoming section, we will explore these methodologies in greater depth, highlighting their contributions to the advancement of RNA biology as well as discussing their individual strengths and potential drawbacks.

## Advances in sequencing technologies for transcriptome-wide mapping of m^6^A

Due to the significant role and prevalence of m^6^A in RNA, many strategies have been devised for m^6^A identification, providing powerful tools to aid study of their biological functions (see Table 1). The majority of these techniques utilize specific antibodies coupled with next-generation sequencing (NGS) to identify m^6^A sites (refer to Sect. “Antibody dependent coupled with NGS sequencing methods”). Initial methods for transcriptome-wide detection offered limited resolution, but subsequent enhancements, such as the integration of crosslinking with advanced bioinformatics—notably the identification of the DRACH motif—have heightened the precision to single-nucleotide resolution. However, the potential bias of antibodies, which may imperfectly discriminate the subtle modifications of m^6^A and their inability to distinguish between m^6^A and its variant m^6^Am, have led to the advent of antibody-independent detection approaches (for further details, see Sect. “Antibody-independent coupled with NGS sequencing methods”). Yet, the integration of such novel methods is often not immediate, resulting in a significant lag between their invention and their practical application (for further details, see Sect. “SARS-CoV-2 as a paradigm for m6A epitranscriptome mapping via sequencing”). This delay probably can be attributed to lower cost, more consistent results, greater familiarity, and enhanced standardization inherent within established techniques. As the saying goes, “the best fit is the best.” Here, we reviewed the main m^6^A detection methods currently available, comparing their advantages and disadvantages. This evaluation aims to assist beginners in selecting the most fitting starting points and research methodologies, keeping in mind that the most suitable choice is often the most effective.

### Antibody dependent coupled with NGS sequencing methods

*The pioneers: MeRIP-seq and m*^*6*^*A-seq*. MeRIP-seq (methylated RNA immunoprecipitation sequencing) [24] marked a significant milestone as the first high-throughput sequencing techniques utilizing m^6^A antibodies. In these methods, mRNA is initially fragmented into 100–200 nucleotides. The fragmented RNA is then incubated with m^6^A antibodies, which specifically bind to m^6^A-modified regions. The eluted RNA is subsequently used for library construction and high-throughput sequencing. Methylated regions are identified as peaks in transcript coverage from immunoprecipitated RNA relative to input RNA, providing a resolution of approximately 200 nucleotides (Table 1). MeRIP-seq and m^6^A-seq are known for their simplicity and have commercialized reagents, making them the initial choices for m^6^A sequencing studies.

*Refined approach: m*^*6*^*A-seq2*. In 2021, a notable advancement was introduced with the development of m^6^A-seq2 [40], an enhanced version of MeRIP-seq/m^6^A-seq. This innovative version streamlines the process by conducting a singular m^6^A immunoprecipitation (m^6^A-IP) on pooled RNA samples, deviating from the traditional one-sample-at-a-time approach. Central to m^6^A-seq2 is the attachment of uniquely barcoded RNA adaptors to fragmented RNA from various samples. By conducting a solitary m^6^A-IP on this mixture, the method distinguishes each sample’s reads by their distinct barcode identifiers. This unification of the immunoprecipitation step into a single reaction vessel significantly curtails technical variability and reduces both the amount of RNA needed and the overall costs associated with library preparation. Furthermore, this consolidated method facilitates a comparative analysis, enabling researchers to assess and contrast global m^6^A modification levels across multiple samples, thus providing deeper insight into the dynamics of m^6^A modifications within a broader biological context.

*Enhancing resolution: PA-m*^*6*^*A-seq*. A UV light cross-linking strategy was introduced for the higher-resolution analysis of the m^6^A modification landscape [37]. 4-thiouridine (4SU) is a photoactive nucleoside analog that can replace uridine in U–A pairing during transcription and improve the efficiency of crosslinking. In photo-crosslinking-assisted m^6^A-seq (PA-m^6^A-seq), 4SU is added to the cell culture, allowing 4SU incorporation into newly synthesized mRNA in place of U; m^6^A-containing RNA is enriched using an antibody, and the RNA is crosslinked with antibodies via UV irradiation (365 nm). Using this method, the researcher mapped m^6^A modifications at a resolution of up to 23 nt. The PA-m^6^A-seq is best suited to detecting m^6^A modifications in cultured cells in vitro and cannot easily be applied on a large scale.

*Advancing to single-base resolution: m*^*6*^*A-CLIP and miCLIP*. The quest for higher resolution led to the development of m^6^A individual-nucleotide-resolution cross-linking and immunoprecipitation methods, such as m^6^A-CLIP and miCLIP [28, 38]. These techniques have successfully generated m^6^A site information with single-base resolution. In m^6^A-CLIP and miCLIP, PA-m^6^A-seq was optimized to remove cell preculture in 4SU. Using this modified approach, fragmented RNA is incubated with m^6^A antibodies, and the RNA–antibody complex is crosslinked using 254 nm UV light. The resulting amino acid residues obtained by protease digestion obstruct the reverse transcription process, resulting in digestion or mutation near the m^6^A site. This process ultimately provides information about m^6^A site location with single-base precision. However, it is essential to note that the efficiency of antibody binding and crosslinking significantly impacts the accuracy of sequencing results. Notably, these methods do not directly identify individual m^6^A sites but rather infer them from the mutation of adjacent pyrimidine sites. This limitation makes it challenging to precisely locate m^6^A in regions with multiple adjacent adenines and analyze the distribution of clustered m^6^A sites.

*Quantifying m*^*6*^*A stoichiometry: m*^*6*^*A-LAIC-seq*. Developed by Molinie et al. [39], m^6^A-level and isoform-characterization sequencing (m^6^A-LAIC-seq) offers a novel approach to m^6^A mapping by directly sequencing full-length transcripts to determine the stoichiometry of m^6^A modifications. Unlike traditional antibody-based mapping techniques, which fragment RNA, this method sequences both antibody-bound and unbound RNA fractions and incorporates External RNA Controls Consortium (ERCC) standards into both immunoprecipitated and supernatant samples. The design of m^6^A-LAIC-seq aims to unravel the complexities of the m^6^A epitranscriptome, uncovering a spectrum of m^6^A levels that vary nonstoichiometrically and are specific to cell type. Additionally, m^6^A-LAIC-seq has uncovered a pronounced tendency for methylated transcripts to associate with proximal alternative polyadenylation (APA) sites, which leads to shorter 3′ untranslated regions, whereas nonmethylated transcripts are more likely to utilize distal APA sites. By circumventing many drawbacks of antibody-dependent methods, m^6^A-LAIC-seq marks a significant advancement in the precise quantification of m^6^A modifications.

### Antibody-independent coupled with NGS sequencing methods

NGS technologies that rely on m^6^A antibodies come with inherent limitations. The consistency of antibody quality is difficult to manage, leading to disparate results when using products from different suppliers. Furthermore, the substantial cost of these antibodies and the considerable quantity of RNA needed for sequencing impede their widespread application. To address these issues, researchers have developed several antibody-independent m^6^A sequencing techniques, offering more uniform and scalable solutions.

*Chemical reactivity-based methods.* Techniques such as m^6^A-SEAL, m^6^A-label-Seq, and m^6^A-SAC-seq emerged to target m^6^A modifications chemically. These techniques, while distinct in their methodologies, collectively strive to enhance the precision and detail of m^6^A site mapping. Among these, m^6^A-SEAL stands out, employing a dual-step chemical process comprising oxidation followed by thiol addition, as outlined by Wang et al. [52]. In this method, the enzyme FTO oxidizes m^6^A to produce an intermediate product, hydroxymethylated m^6^A (hm^6^A), which is subsequently transformed into a stable compound, *N*^6^-dithiolsitolmethyladenosine (dm^6^A), through the application of dithiothreitol. This dm^6^A is then biotinylated to facilitate the enrichment of m^6^A-containing RNA fragments. Subsequent sequencing of this enriched RNA yields detailed m^6^A site information. m^6^A-SEAL is notable for its low RNA input requirements and the robust demethylation capacity of FTO, which exhibits minimal sequence selectivity. However, the technique is not without its limitations, which include a multistep process, the limited catalytic efficiency of FTO, extended duration to completion, and a resolution that is comparable to MeRIP-seq.

m^6^A-label-seq is a metabolic labeling method that feeds the cells with a methionine analog, Se-allyl-l-selenohomocysteine, which can replace m^6^A with *N*^6^-allyladenosine (a^6^A) [29]. The a^6^A positions can be detected using iodination-induced misincorporation during reverse transcription. With this method, the authors demonstrated the detection of 2479 and 2808 m^6^A modification sites in HeLa and HEK293T cells, respectively [29]. However, like m^6^A-SEAL, m^6^A-label-seq can only be applied to in vivo samples. Another method, called selective allyl chemical labeling and sequencing (m^6^A-SAC-seq), streamlines the process by eliminating the cell pretreatment step, according to Hu et al. [53]. This innovative approach directly labels m^6^A sites, encompassing nearly all canonical m^6^A motifs, and quantitatively assesses these sites with single-nucleotide precision. The technique involves the enzymatic addition of an allyl group to m^6^A, producing a^6^m^6^A, which then undergoes cyclization upon iodine treatment. During reverse transcription, reverse transcriptase interprets the cyclized a^6^m^6^A as a mutation. The precise location of m^6^A within the transcriptome is identified by the mutation site, and the mutation rate, compared against a standard curve, provides an accurate measure of m^6^A abundance. m^6^A-SAC-seq holds significant potential for broad application in various biological contexts and shows promise for both foundational research and clinical settings. However, the method’s reliance on an enzymatic reaction introduces a potential bias, as the enzyme may exhibit sequence preferences. Additionally, chemical treatments have been employed to differentiate m^6^A from A based on their tolerability to deamination. Glyoxal- and nitrite-mediated deamination of unmethylated adenosines (GLORI) is the first indirect (such as bisulfite sequencing for DNA 5-methylcytosine), base resolution, and quantitative m^6^A sequencing method [46]. This method is based on nitrous acid mediated adenosine deamination, which had been discovered half a century ago. GLORI has been applied to assesses the m^6^A methylomes of mouse and human cells, unveiling the distribution and stoichiometry of clustered m^6^A modifications [46]. Although GLORI is a method based on chemical reactivity, its efficacy with low-input samples is yet to be determined [54]. Despite this, GLORI has effectively delineated the effects of hypoxia and heat shock on the dynamic alteration of m^6^A modifications [46]. This indicates specific regulatory processes that control m^6^A’s function in gene expression, particularly affecting translation efficiency.

*Enzymatic reactivity-based methods.* Deamination adjacent to RNA modification targets (DART-seq) depends on cytidine deaminase APOBEC1 and m^6^A-binding YTH domain fusion protein to induce C-to-U deamination at sites adjacent to m^6^A modifications [41]. However, this method requires cellular transfection, which limits its application to primary cells and tissue samples. Furthermore, two independent methods: MAZTER-seq [42] and m^6^A-REF-seq [43] used the sensitive MazF RNase, which cleaves RNA only at unmethylated ACA sites, allowing the detection of m^6^A sites at single-base resolution. Both methods provide both base resolution and site stoichiometry. However, they are limited to the subset of m^6^A sites that occur in ACA-containing motifs (i.e., RRACH) and that are located within suitable distances of nearby ACA sequences. In addition, these methods are influenced by the amount of MazF used, as insufficient amounts may result in undigested modified sequence and false site detection. Although these methods can provide single-base resolution mapping of m^6^A sites, their preference for ACA sites means that only around 16–25% of all m^6^A sites across the whole transcriptome can be mapped. Evolved TadA-assisted *N*^6^-methyladnosine sequencing (eTAM-seq) is an enzymatic equivalent of GLORI, which uses a hyperactive transfer RNA adenosine deaminase (TadA) variant TadA8.20 to achieve up to 99% global adenosine-to-inosine deamination [44]. This method employs a hyperactive TadA variant (TadA8.20) for global adenosine-to-inosine deamination, allowing for the transcriptome-wide detection and quantification of m^6^A. It has been used to identify m^6^A sites in HeLa and mouse embryonic stem cells and allows for m^6^A quantification from as few as ten cells [44]. eTAM-seq also aims to preserve RNA integrity and has a much lower input requirement compared with other quantitative profiling methods (see Table 1). Researchers expect eTAM-seq to enable high-resolution m^6^A landscape surveys and detect m^6^A at specific loci with a straightforward workflow (fragmentation, global A deamination, RT–PCR, and Sanger sequencing). However, due to the sensitivity of TadA 8.20 to secondary structures, eTAM-seq requires control transcriptomes to eliminate false positives and may be less accurate at lowly methylated sites (< 25%).

### Mapping m^6^A modifications by third-generation sequencing

In the last decade, TGS technologies represented by two platforms, namely PacBio SMRT and Oxford Nanopore, have emerged as promising alternatives for mapping nucleotide modifications. This section delves into these two pivotal technologies.

*PacBio single-molecule real-time sequencing (SMRT).* The single-molecule real-time (SMRT) sequencing platform by PacBio, a prominent third-generation technology, provides real-time detection of fluorescently labeled nucleotides incorporated during DNA replication from a nonamplified template [48]. Since its commercial release in 2011, SMRT has successfully identified DNA modifications such as 6-methyladenosine (6 mA), 4-methylcytosine (4mC), 5-methylcytosine (5mC), and 5-hydroxymethylcytosine (5hmC). However, the detection of RNA modifications with SMRT sequencing has largely lagged. In 2013, a preliminary method utilizes reverse transcriptase from HIV-1 (Human immunodeficiency virus-1)and Alfalfa mosaic virus (AMV) on a zero-mode waveguide chip indicated the possibility of identifying m^6^A RNA modifications [55]. However, further development in this area has stalled, partly due to the unavailability of the necessary commercial chips, thus limiting SMRT sequencing’s broader application in detecting RNA modifications [56].

*Nanopore direct RNA sequencing (DRS).* Nanopore DRS offers an alternative approach to identifying both DNA and RNA modifications. This technique relies on distinctive alternations on ionic current caused by these modifications, thus eliminating the need for chemical pretreatment typically required in other methodologies [49]. Nanopore DRS discerns modified nucleotides either by contrasting the observed patterns with those of a reference or control sample, or by employing a sophisticated base-calling algorithm trained on dataset inclusive of modified nucleotide information. Notably, research has demonstrated the capacity of Nanopore DRS to identify m^6^A modifications with high accuracy and reduced base-calling performance in modified regions [40, 57–59]. The success of these detections heavily depends on the quality of training datasets that must encompass sequences with and without m^6^A modifications [60]. Despite the potential for these approaches to achieve around 90% accuracy in m^6^A detection, it is important to acknowledge the variability in their effectiveness. For instance, a comprehensive analysis of over ten computational tools designed for mapping m^6^A methylation highlighted the prevalent issue of false positives when relying solely on “errors” to pinpoint modifications. This emphasizes the necessity for control samples with known modification levels to refine detection accuracy [58]. For example, during the COVID-19 pandemic, Nanopore DRS was utilized to map the coronavirus genome, leading to predictions of multiple m^5^C modifications in SARS-Cov-2 [61]. However, the existence of these modifications in SARS-Cov-2 is still controversial, since another study utilizing nanopore sequencing with more rigorous controls did not confirm their presence [62].

In summary, as TGS technologies (i.e., nanopore DRS and SMRT sequencing) continue to advance, the possibility of obtaining maps of RNA modifications such as m^6^A at single-molecule resolution has become a reality [56]. However, as highlighted by Alfonzo et al. [26], these technologies face notable challenges, including high error rates, substantial costs, and stringent sample requirements present significant hurdles. A primary hurdle is the development of robust data analysis software and algorithms. While these technologies are evolving toward greater reliability, they have not yet reached a level of maturity for routine application in RNA epitranscriptomics. Currently, they are often employed alongside traditional methods, mainly serving as supplementary validation tools [58, 59].

### Bioinformatic complexities in m^6^A mapping techniques

Analyzing NGS or TGS data for m^6^A modification studies poses distinct bioinformatic challenges. These challenges arise from the need to accurately identify and quantify m^6^A sites against a complex RNA sequence background. The complexity is further increased by the variable distribution of m^6^A across different RNA molecules and the subtlety of m^6^A-induced changes. To navigate these challenges, researchers must utilize sophisticated computational tools and algorithms tailored for m^6^A detection. Such tools often involve complex processing steps, including adapter trimming, read mapping, peak calling, and data normalization. Moreover, the choice of bioinformatic pipeline can significantly influence the outcomes of m^6^A mapping studies, making the selection of analytical strategies critical [63].

We have reviewed a spectrum of m^6^A detection techniques (Table 1), each of these methods presents its own bioinformatic processing complexities. For instance, techniques such as Nanopore DRS, which provide long-read sequencing capabilities, demand advanced algorithms for accurate m^6^A site identification due to their unique error profiles and data intricacies [58]. Conversely, methods such as MeRIP-seq/m^6^A-seq involve more established bioinformatic workflows but still require careful peak calling and normalization strategies to distinguish true m^6^A signals from background noise [10]. This variability in bioinformatic requirements among the different techniques highlights the need for researchers to not only consider the experimental feasibility of these methods but also the associated bioinformatic challenges. Given the specialized nature of bioinformatic approaches in m^6^A research, we encourage readers seeking detailed insights into the bioinformatic strategies and tools for m^6^A analysis to consult comprehensive review articles in this field, such as [63], which offer extensive discussions on this subject.

## SARS-CoV-2 as a paradigm for m^6^A epitranscriptome mapping via sequencing

Over the decades, the multifaceted role of m^6^A in regulating gene expression, especially its influence on various stages of viral lifecycle and host-virus interactions, has been gradually elucidated [21]. A detailed description of this topic is beyond the scope of this review, and we refer interested readers to some recent comprehensive reviews [11, 12, 16, 22, 64]. In this review, to convey our readers a better understanding of m^6^A sequencing technology in the context of viral infection, we will take the currently most prime and eye-catching virus, SARS-CoV-2, as a paradigm for m^6^A epitranscriptome mapping via sequencing. Recent research has revealed that m^6^A modifications in the SARS-CoV-2 genome play a critical role in regulating viral replication and the host immune response [14–16]. For instance, a study analyzed m^6^A modifications in over 2 million SARS-Cov-2 genomic RNAs from different viral lineages, revealing a potential correlation between the presence of m^6^A modifications and the viral pathogenicity, as well as the effectiveness of vaccines [65].

Until now, there have been 50 published works focusing on m^6^A modifications in the context of SARS-CoV-2 research. Among these, 11 have specifically explored the role of m^6^A in the regulation of viral and host transcriptomes during SARS-CoV-2 infection, employing advanced sequencing technologies as detailed in Table 2. It is noted that the distribution of m^6^A epitranscriptome sequencing technologies in these ten studies actually provides a representative snapshot of their application in contemporary scientific research. Specifically, six studies have utilized MeRIP-seq [14, 66–70], indicating its popularity for m^6^A modification mapping. A single study employed m^6^A-seq [71], and another used miCLIP [65], highlighting the diversity of approaches within the field. Notably, three studies incorporated Nanopore DRS [62, 72, 73], with one instance combining Nanopore with MeRIP-seq [73]. Although research on the application of deep-sequencing technologies in studying SARS-CoV-2 infection so far is limited, the novelty of the virus combined with the rapid accumulation of research within a span of 3 years is still noteworthy. Here we summarized some key insights gained from these closely related studies (Table 2).

**Table 2:** Summary of research on m^6^A modification in SARS-CoV-2 and associated sequencing technologies

Study focus	No. of viral m^6^A peak	Sequencing technologies	Key findings	References
The architecture of SARS-CoV-2 genome inside virion	25	Nanopore DRS	The tertiary structure of the SARS-CoV-2 RNA genome was reconstructed, revealing an “unentangled globule” conformation	[62]
Role of host m^6^A machinery in coronavirus replications	14	MeRIP-seq	Depletion of METTL3 or reader protein could suppress SARS-Cov-2’s replication, indicating the therapeutic potential of targeting the m^6^A pathway to restrict coronavirus reproduction	[66]
Regulation of viral m^6^A RNA modification and host immune response	6–16	MeRIP-seq	SARS-CoV-2 genomic RNA contains m^6^A modifications enriched in the 3′ end region. Depletion of METTL3 decreases m^6^A levels in SARS-Cov-2 and host genes, impacting the innate immune signaling pathway and inflammatory gene expression	[14]
m^6^A epitranscriptome of SARS-CoV-2 genomic RNA	15	Nanopore DRS	m^6^A modification could influence the virus’s ability to evade the immune system and vary among different viral variants	[72]
Role of m^6^A modification in SARS-CoV-2 and how it is modulated by host m^6^A machinery	5 based on MeRIP-seq; 8–14 based on Nanopore sequencing in different cells lines	MeRIP-seq and Nanopore DRS	SARS-CoV-2 RNA contains m^6^A modifications influenced by METTL3. Alterations in METTL3 expression changed the virus’s replication, and the viral protein RdRp was found to interact with METTL3, influencing its distribution and posttranslational modifications	[73]
m^6^A epitranscriptome of SARS-CoV-2 in host cell	8	Combined RIP-seq and miCLIP	SARS-CoV-2 infection triggered a global increase in host m^6^A methylome, exhibiting altered localization and motifs of m^6^A methylation in mRNAs	[15]
Examines how RBM15, an m^6^A methyltransferase, influences COVID-19 severity	N/A	MeRIP-seq	RBM15 promoted the expression of functional genes by elevating m^6^A modification, suggesting RBM15’s modulation of m^6^A modification is a significant factor in COVID-19’s pathogenesis and could be a potential therapeutic target	[68]
Analyze the m^6^A methylome of SARS-CoV-2 and the potential regulatory role of m^6^A in SARS-CoV-2 RNA abundance	11	MeRIP-seq	m^6^A might regulate abundance of SARS-CoV-2 through a mechanism of 3′ UTR with or without RRACH	[67]
Role of FTO during SARS-Cov-2 infections, and how this correlates with the severity of COVID-19 in patients	3	m^6^A-seq	FTO has a significant impact on m^6^A marking on SARS-CoV-2 and may affect the severity of COVID-19	[71]
Exploring how m^6^A RNA modification in host cells is altered during SARS-CoV-2 infection	5	MeRIP-seq	Define the m^6^A modification profile in infected versus uninfected cells, identifying various mRNA and noncoding RNA species with differential m^6^A modification in response to SARS-CoV-2	[69]
Impact of SARS-CoV-2 infection on cellular m^6^A RNA methylation and its subsequent effects on host cell gene expression and stress response mechanisms	Average 10	Refined MeRIP-seq	Infection with various SARS-CoV-2 variants results in a widespread decrease in m^6^A RNA methylation in host cells, disrupting normal cellular processes and stress responses, with variant-specific differences observed in the extent of these effects	[70]

The research landscape surrounding m^6^A sequencing technologies in the context of SARS-CoV-2 infection is diverse and insightful. Liu et al. [15] pioneered the field by profiling the m^6^A methylome in SARS-CoV-2-infected human and monkey cells, discovering a global increase in host m^6^A methylome and widespread m^6^A modifications in the virus’s RNA, indicating its crucial role in the virus’s lifecycle. This was followed by research that delved into the potential of m^6^A machinery as a target for antiviral strategies, finding that inhibition of key RNA methyltransferases METTL3 and m^6^A readers could effectively suppress viral replication [66]. Li et al. [14] explored the interplay between m^6^A modification and the host cell’s innate immune response. They observed that depletion of METTL3 in host cells decreased m^6^A levels in both the virus and host genes, affecting innate immune signaling and inflammatory gene expression [44].

Subsequent analyses demonstrated that SARS-CoV-2 RNA is modified by host–cell m^6^A enzymes, with changes in these enzymes affecting viral replication. A notable interaction between the viral protein RdRp (RNA-dependent RNA polymerase) and METTL3 underscored the intricate relationship between viral replication and host modification mechanisms [73]. Campos et al. [72] utilized Nanopore DRS to identify m^6^A sites in the SARS-CoV-2 genome, revealing about 150 modified bases crucial for function and implicated in the virus’s ability to evade the host immune response. In the context of disease severity, an association was made between the levels of the m^6^A methyltransferase RBM15 and the severity of COVID-19, proposing RBM15 as a potential target for reducing the pathological effects of the virus [68]. Moreover, the role of the RNA demethylase FTO in modulating m^6^A markings on SARS-CoV-2 was also highlighted by Malbec et al. [71], suggesting the enzyme as a therapeutic target based on its influence on disease severity. This year, Vaid et al. [70] reported a global loss of m^6^A in cellular RNAs following SARS-CoV-2 infection, with abundant m^6^A in viral RNA, affecting cellular gene expression and stress responses. More recently, Stacia et al. [69] defined the m^6^A modification profile in SARS-CoV-2-infected cells, identifying various mRNA and noncoding RNA species with differential m^6^A modifications, further enriching our understanding of the virus’s interaction with host cell mechanisms.

In summary, the application of m^6^A epitranscriptome sequencing technologies in SARS-CoV-2 research, as reflected in the ten studies highlighted here, underscores the critical role of m^6^A modifications in viral lifecycle and host immune response. Techniques such as MeRIP-seq, m^6^A-seq, miCLIP, and Nanopore DRS have been instrumental in mapping these modifications, confirming the virus’s ability to modulate the host cell mechanisms and suggesting potential therapeutic targets. The studies collectively contribute to the evolving understanding of the intricate interactions between SARS-CoV-2 and its host, offering insights into the m^6^A’s influence on disease severity and immune evasion, and highlighting the importance of RNA modification in viral pathogenicity.

## Conclusions and prospects

In the past 5 years, the scientific exploration of m^6^A modifications in both host and viral RNAs has undergone significant advancement, illuminating their prevalence and roles during viral infections. This progress has been bolstered by the development of various m^6^A mapping strategies, providing potent tools for probing the biological functions of these modifications (Fig. 2). Despite the availability of high-throughput deep-sequencing techniques offering single-nucleotide resolution, a majority of viral studies have predominantly utilized MeRIP-seq/m^6^A-seq. As previously stated, about 50% of the studies pertaining to SARS-CoV-2 have employed the MeRIP-seq technique (Table 2). The transition to more novel methodologies has been tempered, probably due to factors such as cost-effectiveness, consistency, familiarity, and standardization associated with traditional methods. As we stand on the threshold of new discoveries, it is crucial to acknowledge the ongoing challenges. For instance, transcriptome-wide mapping at base resolution and stochiometric quantification of m^6^A, such as in the recently established GLORI protocol [46], should be pursued to gain further insights into these changes during viral infection. The primary hurdle is the perfection of RNA sequencing technology that is sensitive to modifications [54]. For instance, Nanopore DRS has been applied to the detection of several modifications including m^6^A during viral infections, showing promise for the simultaneous identification of distinct modifications in a single molecule [58, 62]. However, the accuracy and sensitivity are limited [26]. Meanwhile, further innovation is needed to adapt m^6^A mapping for low-input and single-cell samples, which could significantly enrich our comprehension of RNA modifications’ roles in viral infections. Encouragingly, the integration of existing RNA modification detection strategies with single-cell sequencing technologies is showing promise and may catalyze substantial advances in the field [74, 75].

Looking ahead, our research aim should extend beyond mere descriptive analyses to unravel the mechanisms underlying viral RNA modifications and their implications for the viral life cycles [12]. The interplay between host m^6^A machinery and viral infections emerges as a promising area of study. This focus is not only relevant to virology but also to cell biology, given the role of viruses in revealing new facets of cellular processes. Integrating multiomics approaches will be essential in fully comprehending the m^6^A modification landscape during viral infections [76]. Furthermore, targeting these modifications and regulatory proteins could pave the way for novel antiviral strategies. As explored in Sect. “SARS-CoV-2 as a paradigm for m6A epitranscriptome mapping via sequencing,” several studies collectively underscore the significant potential of elements such as METTL3 [14, 66], RBM15 [68], and FTO [71] to serve as potent antiviral targets. Additionally, our recent findings indicate that the microbiome can modulate the host’s tRNA transcriptome in a tissue-specific manner [77], adding a layer of complexity to the interplay between RNA modification pathways, such as m^6^A, with host microbiome interactions.

In conclusion, the exploration of m^6^A modifications in the realm of virology has become increasingly sophisticated since its inception in the 1970s. We are now piecing together a comprehensive picture of how subtle RNA changes impact viral behavior and pathogenesis. The collaboration across scientific disciplines and the fusion of emerging and traditional methodologies are crucial in this endeavor. Far from being a mere academic pursuit, this research has the potential to revolutionize our approach to antiviral therapies, marking a new chapter in the interplay between molecular biology and clinical application.

## Data Availability

No data were used for the research described in the article.

## Abbreviations

m^6^A: *N*^6^-Methyladenosine
COVID-19: Coronavirus disease 2019
SARS-CoV-2: Severe acute respiratory syndrome coronavirus 2
A: Adenine
C: Cytosine
ERCC: External RNA Controls Consortium
G: Guanine
U: Uracil
Ψ: Pseudouridine
m^1^A: *N*^1^-Methyladenosine
m^7^G: *N*^7^-Methylguanosine
m^5^C: 5-Methylcytidine
A-to-I editing: Adenosine-to-inosine editing
IAV: Influenza A virus
MeRIP-seq: Methylated RNA immunoprecipitation sequencing
NGS: Next-generation sequencing
TGS: Third-generation sequencing
2D-TLC: Two'dimensional thin-layer chromatography
LC–MS/MS: Liquid chromatography–tandem mass spectrometry
PA-m^6^A-seq: Photo-crosslinking-assisted m^6^A sequencing
m^6^A-CLIP-seq: M^6^A crosslinking immunoprecipitation sequencing
miCLIP: M^6^A individual-nucleotide resolution UV crosslinking and immunoprecipitation
m^6^A-LAIC-seq: M^6^A-level and isoform-characterization sequencing
DART-seq: Deamination adjacent to RNA modification targets sequencing
m^6^A-REF-seq: M^6^A-sensitive RNA-endoribonuclease-facilitated sequencing
eTAM-seq: Evolved TadA-assisted *N*^6^-methyladnosine sequencing
m^6^A-SEAL-seq: FTO-assisted m^6^A selective chemical labeling method
m^6^A-SAC-seq: M^6^A selective allyl chemical labeling and sequencing
GLORI: Glyoxal- and nitrite-mediated deamination of unmethylated adenosines
RT: Reverse transcription
AIDS: Acquired immunodeficiency syndrome
MERS: Middle East respiratory syndrome
SARS: Severe acute respiratory syndrome
SMRT: Single-molecule real time
PacBio: Pacific Biosciences
ONT: Oxford Nanopore Technologies
4SU: 4-Thiouridine
APA: Alternative polyadenylation
FTO: Fat mass and obesity-associated protein
hm^6^A: Hydroxymethylated m^6^A
dm^6^A: *N*^6^-dithiolsitolmethyladenosine
a^6^A: *N*^6^-allyladenosine
TadA: Transfer RNA adenosine deaminase
6 mA: 6-Methyladenosine
4mC: 4-Methylcytosine
5mC: 5-Methylcytosine
5hmC: 5-Hydroxymethylcytosine
AMV: Alfalfa mosaic virus
DRS: Direct RNA sequencing
RdRp: RNA-dependent RNA polymerase
HIV-1: Human immunodeficiency virus 1
tRNA: Transfer RNA
METTL3: Methyltransferase-like 3
RBM15: RNA-binding motif protein 15
